# The value of CT shape quantification in predicting pathological classification of lung adenocarcinoma

**DOI:** 10.1186/s12885-023-11802-5

**Published:** 2024-01-04

**Authors:** Mingjie guo, Zhan Cao, Zhichao Huang, Shaowen Hu, Yafei Xiao, Qianzhou Ding, Yalong Liu, Xiaokang An, Xianjie Zheng, Shuanglin Zhang, Guoyu Zhang

**Affiliations:** 1 Department of Thoracic Surgery, The First Affiliated Hospital of Henan University, Longting District, 475000 Kaifeng, Henan Province China; 2 Department of Neurology, The Fifth Affiliated Hospital of Zhengzhou University, 450000 Zhengzhou, China; 3 Department of Clinical Medicine, Medical School of Henan University, Kaifeng, China

**Keywords:** Lung adenocarcinoma, CT shape quantification, Pulmonary ground glass nodules, Prediction, Cut-off value

## Abstract

**Objective:**

To evaluate whether quantification of lung GGN shape is useful in predicting pathological categorization of lung adenocarcinoma and guiding the clinic.

**Methods:**

98 patients with primary lung adenocarcinoma were pathologically confirmed and CT was performed preoperatively, and all lesions were pathologically ≤ 30 mm in size. On CT images, we measured the maximum area of the lesion’s cross-section (MA). The longest diameter of the tumor (LD) was marked with points A and B, and the perpendicular diameter (PD) was marked with points C and D, which was the longest diameter perpendicular to AB. and D, which was the longest diameter perpendicular to AB. We took angles A and B as big angle A (BiA) and small angle A (SmA). We measured the MA, LD, and PD, and for analysis we derived the LD/PD ratio and the BiA/SmA ratio. The data were analysed using the chi-square test, t-test, ROC analysis, and binary logistic regression analysis.

**Results:**

Precursor glandular lesions (PGL) and microinvasive adenocarcinoma (MIA) were distinguished from invasive adenocarcinoma (IAC) by the BiA/SmA ratio and LD, two independent factors (*p* = 0.007, *p* = 0.018). Lung adenocarcinoma pathological categorization was indicated by the BiA/SmA ratio of 1.35 and the LD of 11.56 mm with sensitivity of 81.36% and 71.79%, respectively; specificity of 71.79% and 74.36%, respectively; and AUC of 0.8357 (95% CI: 0.7558–0.9157, *p* < 0.001), 0.8666 (95% CI: 0.7866–0.9465, *p* < 0.001), respectively. In predicting the pathological categorization of lung adenocarcinoma, the area under the ROC curve of the BiA/SmA ratio combined with LD was 0.9231 (95% CI: 0.8700-0.9762, *p* < 0.001), with a sensitivity of 81.36% and a specificity of 89.74%.

**Conclusions:**

Quantification of lung GGN morphology by the BiA/SmA ratio combined with LD could be helpful in predicting pathological classification of lung adenocarcinoma.

## Introduction

Lung cancer continued to be the most common cause of cancer-related deaths, according to a previous report [1]. Due to the widespread use of high-resolution thin-layer CT in physical examinations and the extensive implementation of lung cancer screening in high-risk groups, detection rates have improved significantly in recent years [2, 3]. Depending on whether solid components were present or not, ground glass nodules (GGN) might be categorized as pure ground glass nodules (pGGN) or mixed ground glass nodules (mGGN). Lung GGN could be either benign lesions or early or progressive stages of malignant lesions [4]. Among them, the infiltrative lesions of lung adenocarcinoma-associated GGN progressed faster and were prone to metastasis, affecting the survival and prognosis of patients. Lung GGN was found in any stage of lung adenocarcinoma [5], such as invasive adenocarcinoma (IAC), microinvasive adenocarcinoma (MIA), adenocarcinoma in situ (AIS), and atypical adenomatous hyperplasia (AAH). AAH and AIS were designated as precursor glandular lesions (PGL) in the WHO Classification of Thoracic Tumors (5th edition), which was released by the International Agency for Research on Cancer (IARC) in 2021 [6].

It was difficult to determine the aggressiveness of the tumor before and during surgery due to the obvious limitations in determining the aggressiveness of the tumor in the tissue specimens obtained from the intraoperative rapid frozen Sect. [7]. Imaging-based 3D measurements of tumor size changes may provide a more accurate and direct assessment of the treatment course of tumors [8]. Computed tomography (CT) is widely used in clinical practice for tumor detection, staging and monitoring of treatment response [9, 10]. With the rapid development of imaging technology in clinical application, the analysis of CT radiological parameters could be used for clinical decision-making and provided an accurate diagnostic tool [11]; moreover, the assessment of lung adenocarcinoma aggressiveness using preoperative imaging features showed great advantages and became a global research hotspot in recent years [12].

Lung GGN suspected of being malignant in clinical practice was still predominantly treated with surgery. In PGL and MIA, the application of anatomical segmental resection and wedge resection had a similar prognosis to that of radical lung cancer surgery and maximized the preservation of lung function [13–15], patients with negative surgical margins had a 5-year survival rate of 100% [16]. Nonetheless, there was a larger likelihood of IAC associated with lymph node metastases, and the prognosis for patients was poorer [17]. Therefore, meticulous lymph node dissection along with radical lung cancer surgery was needed. In view of the different treatment options and prognosis of PGL and MIA from IAC, this study proposed to quantify the shape of lung GGN associated with lung adenocarcinoma and analysed the quantitative parameter information to investigate its clinical value for predicting PGL and MIA from IAC, which will provide some reference for the preoperative evaluation of the patients and the selection of treatment options.

## Materials and methods

### Clinical information

**Fig. 1:**
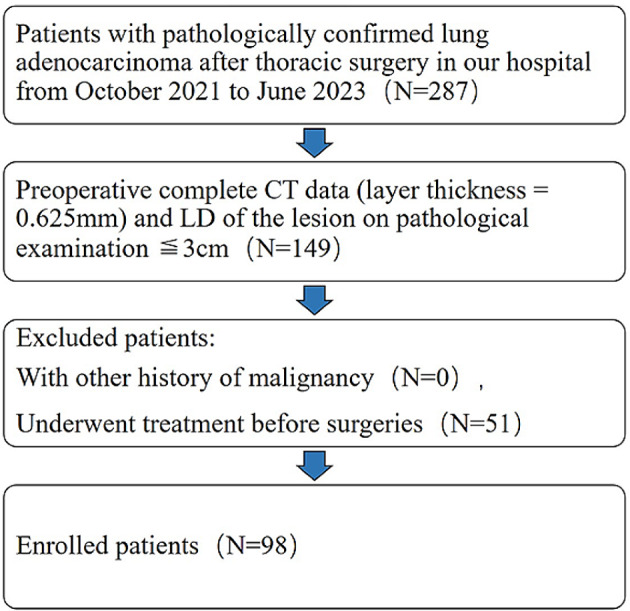
Flowchart that illustrates the inclusion procedure. LD, the longest diameter

From October 2021 to June 2023, the clinical and CT imaging data of 98 patients with lung adenocarcinoma presenting as GGN on CT confirmed by surgical pathology in the First Affiliated Hospital of Henan University were retrospectively studied. The study’s inclusion criteria were as follows: (1) postoperative pathological diagnosis of lung adenocarcinoma and a single lesion, with the largest diameter of the tumor ≤ 3 cm; (2) preoperative chest CT lesion images showing GGN; (3) thin-layer CT images (layer thickness = 0.625 mm) were available preoperatively. The exclusion criteria for the study were as follows: (1) patients who had other malignant tumors in the past; (2) those who had received treatment for the relevant tumor prior to preoperative scanning CT; (3) Pathological examination showed that the size of the lesion was > 3.0 cm. Figure 1 showed the workflow diagram for patient selection in the study. Ultimately, a total of 98 patients were included (98 lesions in total): 37 males (mean age 58.4 ± 1.73 years; range 40–83 years) and 61 females (mean age 57.8 ± 11.18 years; range: 29–77 years). According to the degree of infiltration diagnosed by pathology, they were divided into 2 groups: (1) PGL and MIA group: a total of 39 patients (39 GGN lesions), including 13 males and 26 females, age range: 29–73 years old, mean age 54.4 ± 12.1 years. (2) IAC group: a total of 59 patients (59 GGN lesions), of which 24 were male and 35 were female, age range: 39–83 years old, mean age 60.4 ± 9.4 years.

### Acquisition of CT images

All 98 patients underwent non-contrast CT of the chest or non-contrast CT enhancement of the chest. High-speed spiral CT scanners (GE Healthcare) with 128-slice or 64-slice were used for all CT scans. The patient’s position was supine, and a CT scan of the whole chest was conducted at the end of inspiration and after breath-holding, ranging from the apex to the base of the lung. The following parameters were applied to the CT scan: tube voltage of 120 kV, tube current of 150–200 mA, pitch of 0.984:1, rotation speed of 0.5 s/r, and layer thickness of 0.625 mm. The images were reconstructed with hybrid iterative reconstruction algorithm, contiguous 0.625-mm-thick slices, and the lung window was adjusted to have a window width of 1,500 HU and a window level of -450 HU. All enrolled images were axial plain CT images.

### Image analysis

All patient CT scan image data were transferred to PACS and Mimics for storage and analysis. Two senior physicians with over six years of expertise in reading images were employed to measure the GGN lesions of the research cases in the transverse, coronal, and sagittal planes using the double blind experimental method. The following parameters were measured: the maximum area of the lesion’s cross-section (MA), the longest diameter (LD), and the greatest perpendicular diameter of the longest diameter (PD). In case of disagreement, the opinion was harmonized after consultation or the involvement of a senior imaging physician in the assessment.

**Fig. 2:**
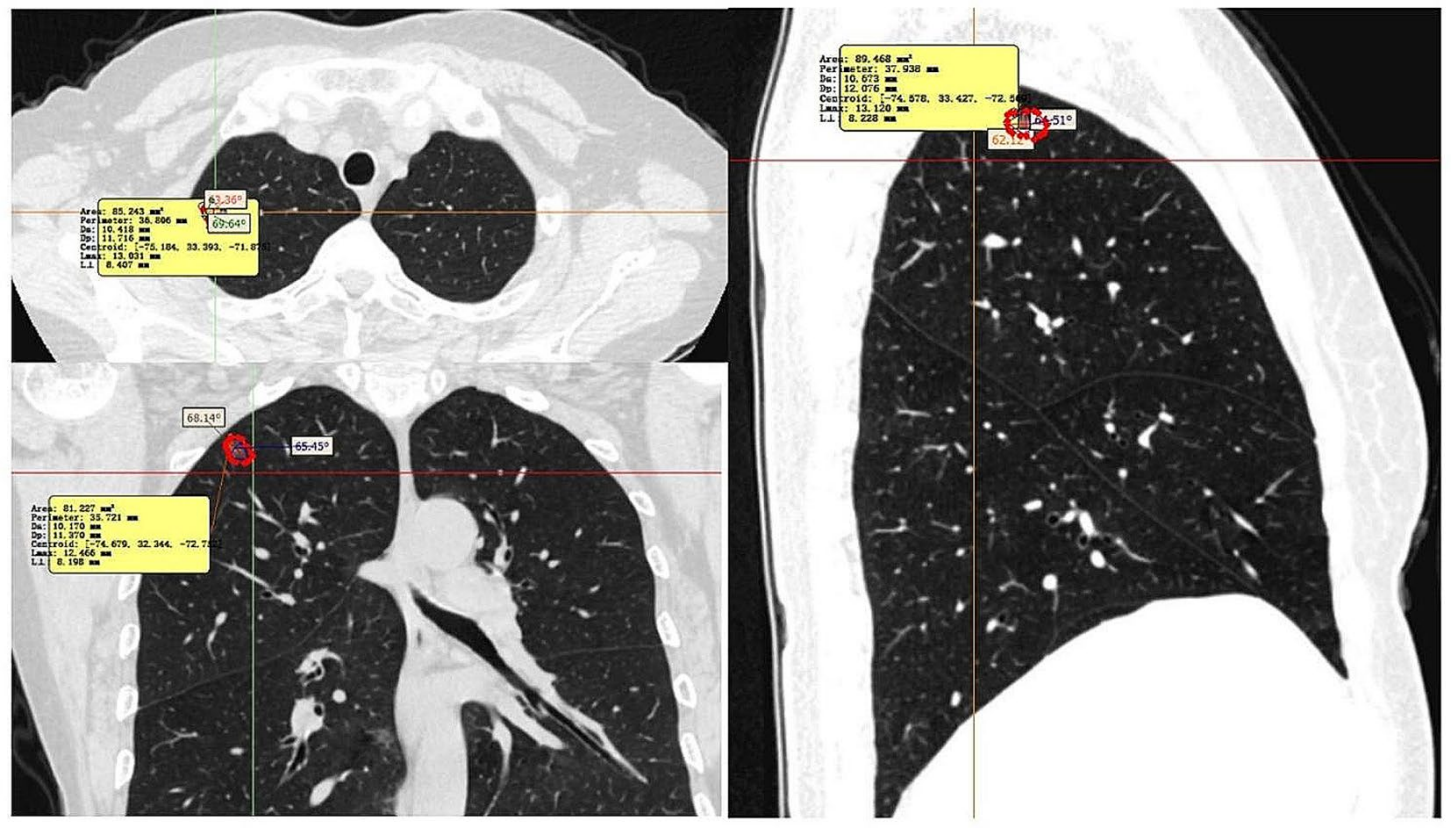
Comparison of the lung GGN’s axial, coronal, and sagittal imaging measurements

**Fig. 3:**
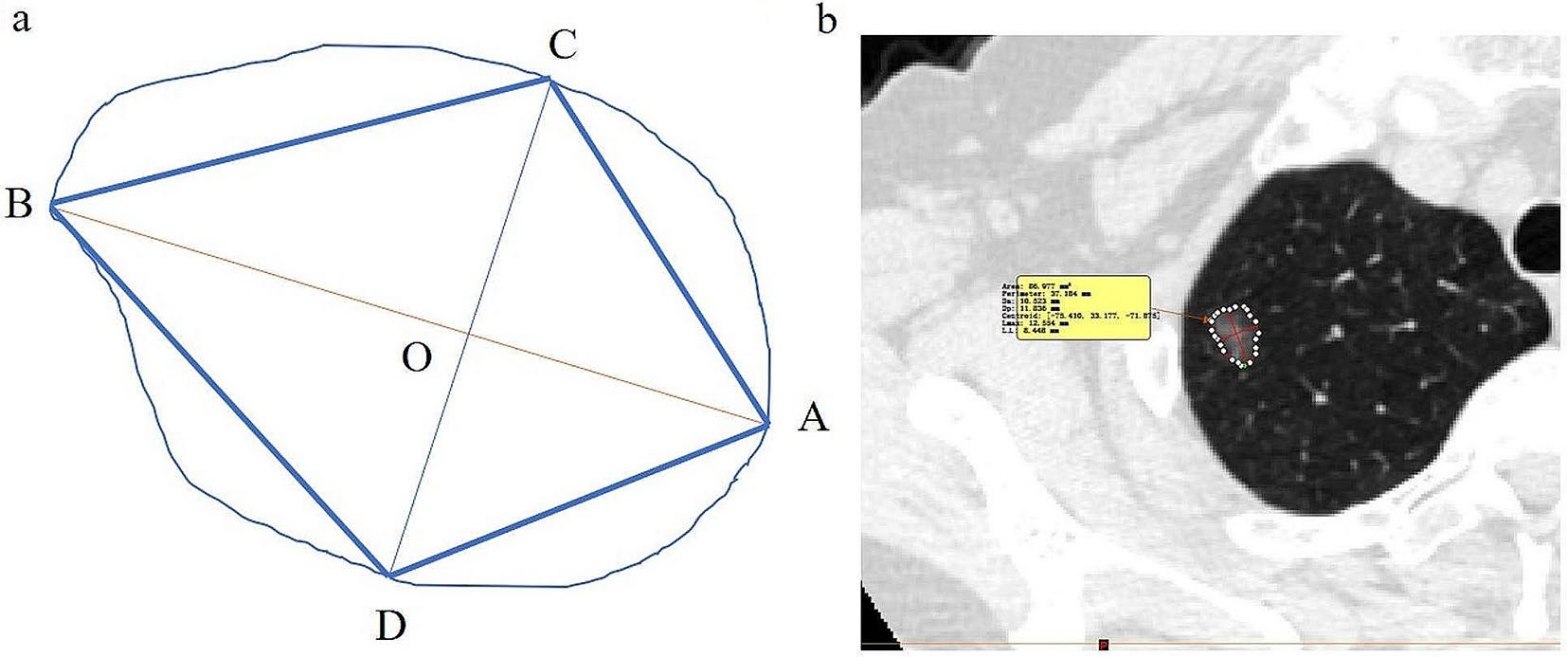
Two-dimensional schematic diagram and actual tumor measurement diagram of GGN. (**a**) Two-dimensional schematic diagram, we chose angle A (77.03°) and angle B (60.55°) as the big angle (BiA) and the small angle (SmA), respectively. Then, we obtained the BiA/SmA ratio (77.03°/60.55°), which was approximately 1.2722. (**b**) Actual tumor measurement image

When determining the LD and PD of the lesion in transverse, coronal, and sagittal multiplanar reformation (MPR), the largest cross-section of the lesion was chosen (Fig. 2). After obtaining the maximum cross-section, the cross-section was carefully outlined with Mimics to obtain MA, LD (line “AB”) (Fig. 3) was the maximum diameter, and the maximum diameter perpendicular to LD was PD (line “CD”), and ABCD could be formed into a quadrilateral. Subsequently, the quadrilateral ABCD’s four angles were measured, with each angle being identified by its vertex (A, B, C, D). The lines AB and CD intersected at point O, and the angles that corresponded to LD were angle A (Big angle, BiA) and angle B (Small angle, SmA) (∠A ≥∠B). We explained the lesions in detail by using pattern diagrams (Fig. 3a). In Fig. 3a, LD and PD intersect at point O. The nearer point A is to point O, the larger the angle A is. The larger the ∠A/∠B ratio, the more pronounced the growth of the tumor in the direction of “OB”, and the more irregular the entire mass. Two authors independently measured each lesion’s MA, LD, PD, BiA and SmA, and the LD/PD ratio and BiA/SmA ratio were computed. Figure 3b is an actual tumor measurement image.

### Statistical analysis

In our study, we divided the adenocarcinoma pathology into PGL and MIA group and IAC group according to the degree of infiltration diagnosed by the pathology. We employed the chi-square test for categorical data, the t-test or nonparametric test for continuous variables, and the MA, BiA, BiA/SmA ratio, LD, and LD/PD ratio to determine whether there were statistically significant differences between the previously indicated two groups. A difference could be considered statistically significant if the p-value was less than 0.05. In order to determine the independent factors affecting the pathological classification of adenocarcinomas, a binary logistic regression analysis was carried out. The BiA/SmA ratio and LD were found using receiver operating characteristic (ROC) analysis, which also allowed us to differentiate the optimal cut-off values for the PGL and MIA group and the IAC group. The optimal cut-off value was the sum of sensitivity and specificity minus 1. The outcomes were sorted in an Excel table to obtain the Youden index, which represents the greatest value of the ideal cut-off value. Then, the specificity and sensitivity corresponding to it were obtained. In addition, we used the predictive probability equation of the logistic regression model to simulate the combined diagnosis of BiA/SmA ratio and LD, which was analysed using the above method to predict its value in determining the pathological staging of lung adenocarcinoma.

## Results

**Fig. 4:**
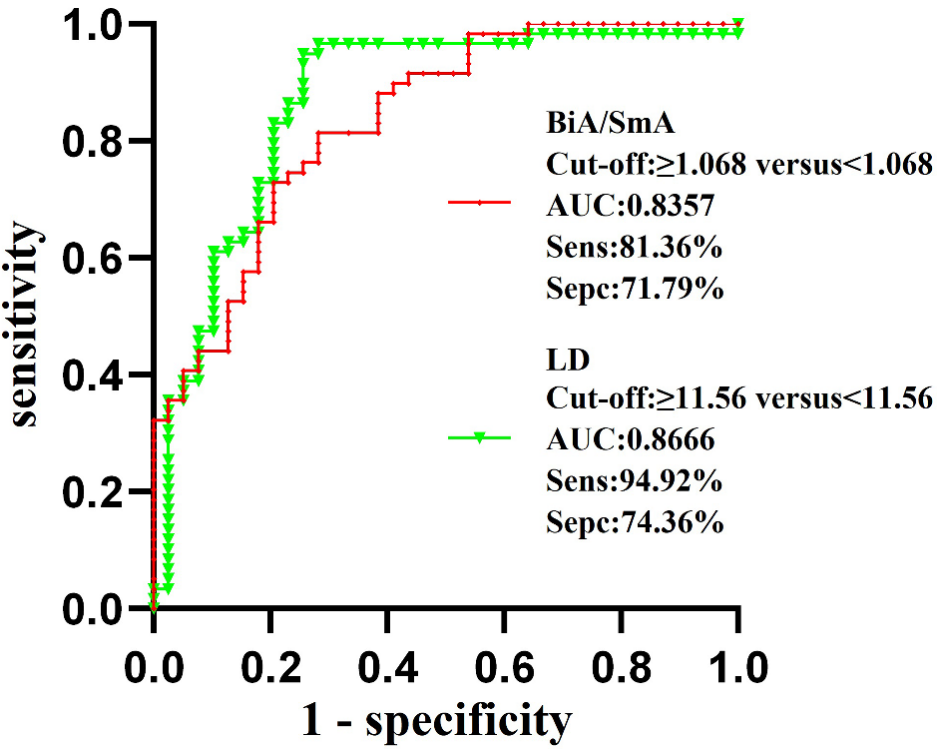
The receiver operating characteristic curve for both LD and the BiA/SmA ratio. AUC, area under the curve; Sens, sensitivity; Spec, specificity

**Fig. 5:**
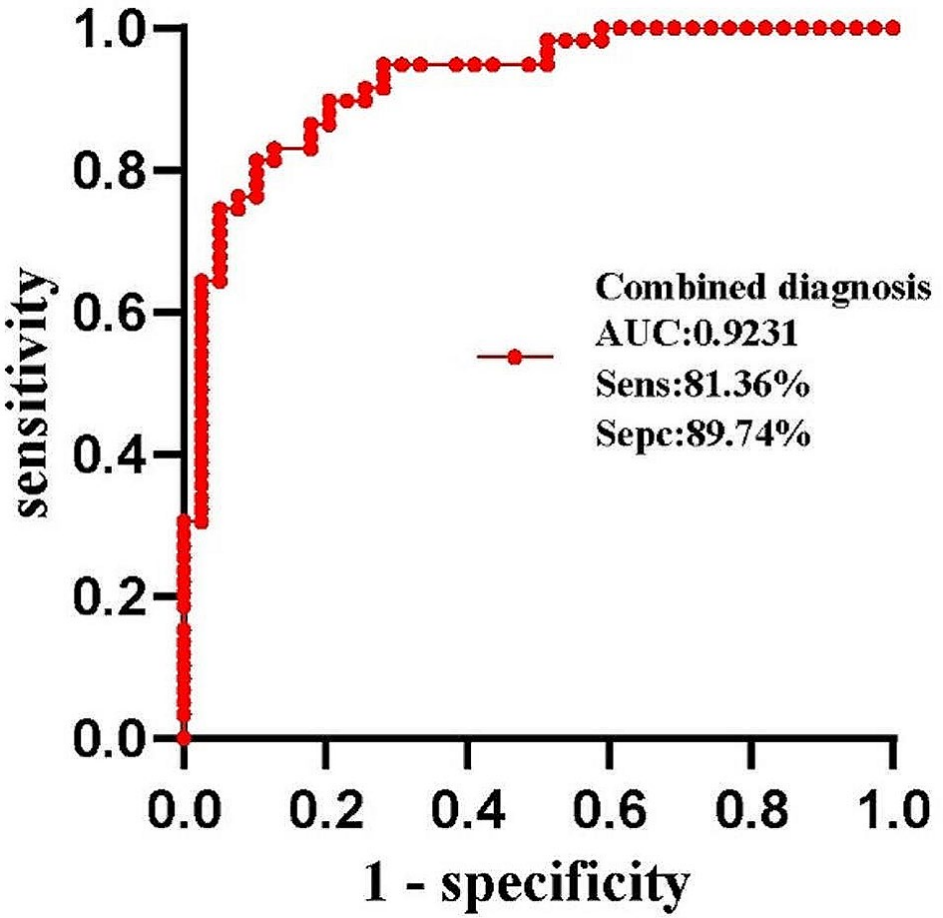
Receiver operating characteristic curve for lung pathologic categorization of adenocarcinoma using the combination of LD and the BiA/SmA ratio. AUC, area under the curve; Sens, sensitivity; Spec, specificity

Table 1 displayed the GGN and basic clinical data for the 98 participants included in this investigation, in which the P-values for sex, age and nodule location were 0.527, 0.079 and 0.699, respectively, and none of these indicators were statistically significant (*P* > 0.05). In addition, 43.6% (*N* = 17) of the nodules in the PGL and MIA groups had their location in the upper lobe of the right lung, 42.4% (*N* = 25) of the nodules in the IAC group had their location in the upper lobe of the right lung, and our statistics showed that the location of the nodules was in the upper lobe of the right lung in 42.9% of the total number of cases (*N* = 42). The mean values, standard deviation, and p-values of MA, BiA, BiA/SmA ratio, LD, and LD/PD ratio in both groups were shown in Table 2. Among them, all statistical indicators were higher and statistically different in the IAC group than in the PGL and MIA groups (*p* < 0.05). The BiA/SmA ratio and LD were found to be independent factors influencing their pathological classification using binary logistic regression analysis. Additionally, the BiA/SmA ratio and LD were included in the predictive probability equation of the logistic regression model. This logistic regression model’s predictive probability equation P = e^− 34.571+28.443 × 1+0.27 × 2^/1 + e^− 34.571+28.443 × 1+0.27 × 2^, where the variable X1 represented the BiA/SmA ratio (OR = 2.253e^12,95%CI: 959666.879-5.289e^^18^, *P* < 0.001)and variable X2 represented LD (OR = 1.310, 95% CI: 1.155–1.485, *P* < 0.001). The ROC curves for the BiA/SmA ratio and LD, shown in Fig. 4, showed the area under the curve (AUC), were 0.8357 (95% CI: 0.7558–0.9157, *p* < 0.001), 0.8666 (95% CI: 0.7866–0.9465, *p* < 0.001), respectively. The BiA/SmA ratio had a sensitivity of 81.36% and a specificity of 71.79%, which corresponded to a Youden index of 1.068; the LD had a sensitivity of 94.92% and a specificity of 74.36%, which corresponded to a Youden index of 11.56. Figure 5 displayed the ROC curve for the combination of BiA/SmA ratio and LD in predicting the pathological classification of lung adenocarcinoma. The curve had an area under the curve of 0.9231 (95% CI: 0.8700-0.9762, *p* < 0.001), a sensitivity of 81.36% and a specificity of 89.74%.

**Table 1:** Basic information of GGN in 98 patients

	PGL and MIA	IAC	P value
Sex			0.527
Men	13(33.3%)	24(40.7%)	
Women	26(66.7%)	35(59.3%)	
Age	54.4 ± 12.1	60.4 ± 9.4	0.079
Position			0.699
Superior lobe of right lung	17(43.6%)	25(42.4%)	
Middle lobe of right lung	4(10.3%)	4(6.8%)	
Inferior lobe of right lung	4(10.3%)	8(13.6%)	
Upper lobe of left lung	8(20.5%)	17(28.8%)	
Inferior lobe of left lung	6(15.4%)	5(8.5%)	

**Table 2:** Analysis of CT quantitative parameters in two groups

	PGL and MIA	IAC	P value
MA(mm^2^)	97.029 ± 110.315	224.752 ± 154.947	0.005
LD(mm)	11.818 ± 4.742	19.488 ± 6.060	0.005
BiA	80.315 ± 6.763	74.617 ± 12.477	0.001
LD/PD	1.247 ± 0.161	1.491 ± 0.568	0.022
BiA/SmA	1.055 ± 0.042	1.136 ± 0.080	< 0.001

The data was displayed as mean ± standard deviation, with *P* < 0.05 denoting statistical significance

## Discussion

It has been demonstrated that pulmonary GGN is associated with pathological aggressiveness, and assessment of its aggressiveness can guide clinical treatment with some clinical significance [18]. Previously, there were several studies on the CT morphology of pulmonary ground-glass nodules, including the air-bronchial sign, vessel penetration sign, vacuolar sign, burr sign, pleural pulling sign, etc. [19–22]. However, these morphological analyses were susceptible to subjective factors and lacked clear objective indicators. The CT quantitative data were more objective and direct, allowing direct quantitative analysis of GGN with parameters that are important for predicting lung pathological subtypes [23, 24].

Tumor cells in lung GGN usually grow in a squamous pattern along the original alveolar structure [25]. Maximum cross sections could be easily obtained, quantified into quadrangles, and measured for their maximum diameter and angle using some of the measurement tools in PACS and Mimics. It may be possible to differentiate between benign and malignant lung cancers using form features that were extracted by computers [26]. A solid tumor needs to be able to withstand the ensuing compressive force to grow in a limited region defined by surrounding tissue [27]. According to Cheng et al., a strong correlation was found between the distribution of solid stress around the ellipsoid and the shape of the ellipsoid, which is due to the inhibition of cell proliferation and the induction of apoptotic cell death in areas of high mechanical stress [28]. Tumor morphology can differentiate between several pathogenic types of breast cancer, according to Yoon et al. [29]. According to Okabe et al., irregular tumor shape identified by preoperative computed tomography was associated with a poor prognosis following aggressive surgery for neuroendocrine tumors of the pancreas [30]. Wei et al. found a strong relationship between the degree of risk and the shape of GIST [27]. Therefore, we considered that there was a correlation between the shape of the lung GGN and the pathological classification of lung adenocarcinoma.

As shown in Table 1, the clinical factors of sex, age and nodule location had no effect on our results (*P* > 0.05). In our study we also found that lung nodule location was highest in the PGL and MIA group and IAC group located in the upper lobe of the right lung. In this study, we verified that the BiA/SmA ratio and LD were independent factors for distinguishing PGL and MIA from IAC by binary logistic regression analysis. The ROC analysis of the BiA/SmA ratio and LD showed that when the BiA/SmA ratio ≥ 1.068 and LD ≥ 11.56 mm, the pathological subtypes of lung adenocarcinoma corresponding to lung GGN were more inclined to IAC. The combination of the BiA/SmA ratio and LD had better value in predicting pathological classification. In the best of our understanding, there were no previous reports of lung GGN shape quantification in the angular prediction of lung adenocarcinoma pathologic classification. In our study, we achieved this by converting qualitative analysis into quantitative analysis of images in PACS and Mimics using CT 3D reconstruction imaging.

This research had a number of limitations. First, the design of this study was retrospective and selection bias might have occurred, for example, only surgically resected GGNs were included, whereas some GGNs followed up annually were not included because there was no pathological diagnosis. In addition, due to the data were from a single center, the sample size was relatively small and more clinical centers would need to be involved. Next, because of the poor contrast between pulmonary GGN and the surrounding lung parenchyma, and because some peripheral GGN have vascular penetration signs and pleural pulling signs, quantitative GGN data measured manually may not be very accurate, and automated methods of measurement are not yet well realized. In this study, the measurements were taken using the lung window, which has a window width of 1500 HU and a window level of -450 HU [25]; however, the GGN includes both the pGGN and mGGN, and different window widths and window levels may affect the measurements [31, 32]. Consequently, it was essential to confirm our assessment through additional research.

## Conclusions

In summary, shape quantification of lung GGN was performed, and the quantitative data BiA/SmA ratio and LD were independent influences to differentiate PGL and MIA from IAC, and BiA/SmA ratio ≥ 1.068 and LD ≥ 11.56 mm could differentiate PGL and MIA from IAC. Moreover, the combined BiA/SmA ratio and LD for the prediction of pathological staging of lung adenocarcinoma had better diagnostic efficacy.

## Data Availability

The datasets used and analysed during the current study are available from the corresponding author on reasonable request.

## Abbreviations

GGN: Ground glass nodules
pGGN: Pure ground glass nodules
mGGN: Mixed ground glass nodules
IAC: Invasive adenocarcinoma
MIA: Microinvasive adenocarcinoma
AIS: Adenocarcinoma in situ
AAH: Atypical adenomatous hyperplasia
PGL: Precursor glandular lesions
LD: The longest diameter
MA: The maximum area
PD: The greatest perpendicular diameter of the longest diameter
BiA: Big angle A
SmA: Small angle A
ROC: Receiver operating characteristic
AUC: Area under the curve
Sens: Sensitivity
Spec: Specificity
